# ONT long-read WGS for variant discovery and orthogonal confirmation of short read WGS derived genetic variants in clinical genetic testing

**DOI:** 10.3389/fgene.2023.1145285

**Published:** 2023-04-21

**Authors:** Ludmila Kaplun, Greice Krautz-Peterson, Nir Neerman, Christine Stanley, Shane Hussey, Margo Folwick, Ava McGarry, Shirel Weiss, Alexander Kaplun

**Affiliations:** Variantyx Inc, Framingham, MA, United States

**Keywords:** long read sequencing, structural variants (SVs), orthogonal variant confirmation, clinical genetic testing, whole genome sequencing (WGS), oxford nanopore technologies (ONT), small sequence changes (SSCs)

## Abstract

Technological advances in Next-Generation Sequencing dramatically increased clinical efficiency of genetic testing, allowing detection of a wide variety of variants, from single nucleotide events to large structural aberrations. Whole Genome Sequencing (WGS) has allowed exploration of areas of the genome that might not have been targeted by other approaches, such as intergenic regions. A single technique detecting all genetic variants at once is intended to expedite the diagnostic process while making it more comprehensive and efficient. Nevertheless, there are still several shortcomings that cannot be effectively addressed by short read sequencing, such as determination of the precise size of short tandem repeat (STR) expansions, phasing of potentially compound recessive variants, resolution of some structural variants and exact determination of their boundaries, *etc.* Therefore, in some cases variants can only be tentatively detected by short reads sequencing and require orthogonal confirmation, particularly for clinical reporting purposes. Moreover, certain regulatory authorities, for example, New York state CLIA, require orthogonal confirmation of every reportable variant. Such orthogonal confirmations often involve numerous different techniques, not necessarily available in the same laboratory and not always performed in an expedited manner, thus negating the advantages of “one-technique-for-all” approach, and making the process lengthy, prone to logistical and analytical faults, and financially inefficient. Fortunately, those weak spots of short read sequencing can be compensated by long read technology that have comparable or better detection of some types of variants while lacking the mentioned above limitations of short read sequencing. At Variantyx we have developed an integrated clinical genetic testing approach, augmenting short read WGS-based variant detection with Oxford Nanopore Technologies (ONT) long read sequencing, providing simultaneous orthogonal confirmation of all types of variants with the additional benefit of improved identification of exact size and position of the detected aberrations. The validation study of this augmented test has demonstrated that Oxford Nanopore Technologies sequencing can efficiently verify multiple types of reportable variants, thus ensuring highly reliable detection and a quick turnaround time for WGS-based clinical genetic testing.

## 1 Introduction

Rapid evolution of the DNA sequencing technologies encourages a paradigm shift in clinical genetic testing from phenotype driven tests focused on particular genes or panels to a comprehensive and rapid approach, aimed at minimizing the time from symptom detection to diagnosis and treatment, and at the same time reducing the patient distress and overall cost of healthcare. Whole Genome Sequencing (WGS) allows such comprehensive testing with simultaneous discovery of most types of clinically relevant genetic variants, reducing the diagnostic odyssey to a one step process. In 2021 a bill was introduced in the US Senate, intended to improve access to WGS services for children with suspected genetic or undiagnosed disease with a goal to achieve better clinical outcomes (Collins, 2021). Recently, a multi-hospital randomized clinical trial has demonstrated medical advantages of the WGS diagnostic approach in terms of change of management, diagnostic efficacy, mortality, *etc.* (Krantz et al., 2021). Multiple medical institutions have already implemented either an in-house or outsourced access to the WGS-based diagnostics for their patients, including the “Project Baby Bear” (coordinated by California state program) (Dimmock et al., 2021), Precision Health programs by Stanford Medicine and Yale Medicine (Stanford Medicine, 2021; Yale Medicine, 2023) *etc.* It should be noted that the majority of WGS sequencing tests offer short read technology, as it has been the longest on the market, hence providing well established analytical tools and multiyear efforts invested in process refinement and cost reduction. Despite those benefits, due to its inherent technical limitations, short reads sequencing cannot provide a definitive resolution for some types of variants such as structural variants with exact detection of boundaries, precise size detection of expanded short tandem repeats, phasing of potentially compound variants, *etc.* In addition, some regulatory authorities may require an orthogonal confirmation of all reportable variants regardless of their type (New York State. Department Health., 2020). All that necessitates an involvement of additional diagnostic or confirmatory solutions (such as Southern blot, microarray, qPCR, *etc.*), hence impeding the diagnostic process, increasing costs especially when multiple variants and variant types are involved, compounding the logistics, and making the diagnostic process as a whole inefficient and error prone. At Variantyx we have developed a universal orthogonal confirmation system utilizing long reads sequencing performed with Oxford Nanopore Technologies (ONT) approach (Oxford Nanopore Technologies, 2023b) for verification of all variants detected by the initial short reads sequencing. Comprehensive approach combining in house short read- and long read-based approaches minimizes the time to diagnosis, enabling combinatorial variant inspection and verification by clinical interpretation specialists, providing thoroughly confirmed results, and strictly adhering to the CLIA guidelines for clinical genetic testing laboratories (Centers for Medicare and Medicaid Services, 2023).

Utilization of long reads sequencing as a standalone WGS clinical diagnostic platform is being currently attempted utilizing ONT long reads (Gorzynski et al., 2022) and PacBio Hi-Fi technology (Sanford Kobayashi et al., 2022), however it is still significantly hindered by high cost, relatively low throughput (particularly of PacBio-based sequencing), and additional limitations, such as, for example, imprecise basecall accuracy of the ONT-based method (Marx, 2023). Our approach allows combination of the best of both WGS methods, combining the variant detection by short reads with results confirmation and additional information supplied by the relatively low pass (12x) long reads sequencing. Implementation of such supplementary long reads WGS testing is not only designed for reporting highly reliable orthogonally verified results, but also enhances genetic testing with improved resolution of multiple variant types, and enables future improvement of the analytical approaches, expanding into additional testing fields not currently accessible to short reads technologies, such as epigenetic modification, immunogenetics analysis, *etc.* This approach that allows to achieve better results by augmenting the capabilities of short read technologies with long read strength are apparent to a broad scientific community and is currently being implemented for research and referencing purposes by Telomere-to-Telomere Consortium (T2T) (Nurk et al., 2022), Human Pangenome Reference Consortium (HPRC) (Wang et al., 2021) *etc.*

## 2 Materials and methods

### 2.1 Samples and truth sets

Twenty eight samples have been acquired as gDNA from Coriell repository (Coriell Institute, 2023), including two well characterized Genome In A Bottle (GIAB) samples with available truth sets (Diroma et al., 2014; Parikh et al., 2016; Krusche et al., 2019; Zook et al., 2016; Zook et al., 2020; Davis et al., 2022; Sayers et al., 2022; NIST, 2023) and twenty six samples with known pathogenic variants as indicated at Coriell repository sample characteristics. Additional fifteen gDNAs were extracted from de-identified clinical samples (labeled Sample_1 … Sample_15) carrying pathogenic variants that were previously orthogonally tested when applicable. All clinical samples were collected with appropriate signed informed consents. See summary of all utilized samples in Supplementary Table S1.

Sample collection has been performed with Lavender K2-EDTA Blood Collection Tubes (367861, PulmoLab, Northridge, CA) and with saliva collection tubes (OGD-500 and OCR-100, DNAGenotek, Kanata, ON, Canada).

### 2.2 Library preparation and sequencing

1,500 ng gDNA has been sheared with G-Tubes utilizing standard 10 kb protocol (SKU520079, Covaris LLC, Woburn, MA). The resulting DNA fragments were used for library preparation utilizing standard ONT Ligation sequencing gDNA protocol with multiplex ligation sequencing kit 11 (SQK-MLK111, ONT, Oxford, UK) with incorporated native barcoding and low molecular weight fragment elimination buffer. Samples were sequenced using PromethION P-24 ONT device (ONT, Oxford, UK (Oxford Nanopore Technologies, 2023c)) with R9.4 flow cells (FLO-PRO002, ONT, Oxford, UK). Only flow cells with at least 5,000 pores at the initial scan were used for sequencing, and at least 2,500 were expected to be still available 24 h after the beginning of the run as a quality assurance parameter of stable performance.

WGS sequencing has been performed with three samples per flow cell for 72h, utilizing additional sequencing in cases where not enough data has been accumulated for certain samples due to DNA quality limitations or other technical issues. Raw data collection was performed with a quality score above 9 (average ∼16, Supplementary Figure S2), at a pore translocation speed above 300 bases/sec for at least 75% of the pores, and stored in fast5 data files, with basecalled files output is fatsq for further processing.

### 2.3 Bioinformatics

Basecalling on High Accuracy Settings (HAC) and demultiplexing has been performed in parallel with sequencing by MinKnow v22.08.6 software (Oxford Nanopore Technologies, 2023a) integrated with PromethION P-24 sequencing device. The acquired reads were processed with Variantyx proprietary Genomic Intelligence platform (https://www.variantyx.com/), combining publicly available bioinformatics tools (Minimap2 v.2.24 (https://github.com/lh3/minimap2), Mosdepth v0.2.8 (https://github.com/brentp/mosdepth), Clair3 v0.1 (https://github.com/HKU-BAL/Clair3), CuteSV v1.0.10 (https://github.com/tjiangHIT/cuteSV) with internally developed proprietary software. Genome assembly hg38 (Genome Reference Consortium, 2017) has been used as a reference. Variantyx intelligence platform is designed for data processing from raw signal to genetic interpretation and report generation, allowing enhanced reliability of variant detection and verification. The platform is designed to process both short-read sequencing data (utilized for primary variant detection), and long-read sequencing data (utilized for orthogonal verification) with convenient transition between both sets of the results. That platform includes a proprietary diagnostic console with integrated accessibility of information available through curated outside databases and internal knowledge base, as well as proprietary visualization aid displaying deviations in allele fraction and depth to support discovery of large structural events. This console allows a user to quickly access the variants already detected by other methods and visualize them for inspection either manually or with additional supporting tools.

### 2.4 Analytical approach

Due to a suboptimal raw read accuracy yielded by the ONT currently released flow cell (R9.4) and chemistry versions (Oxford Nanopore Technologies, 2023d) (see Supplementary Figure S2; Supplementary Table S4), leading to the reduced quality of variant calling, careful visual inspection of all analyzed variants has been implemented as a safeguard against false results. For the validation process, previously documented variants from Genome in a Bottle (GIAB) truth sets and clinically relevant variants previously confirmed by other techniques were used. Known coordinates of the variants in question were utilized for inspection of the appropriate locations in the long read sequencing results. Analytical approach adhering to the same procedure with inspection of appropriate chromosomal coordinates for orthogonal verification of the variants detected using short reads WGS has since been implemented at Variantyx for routine clinical use (Supplementary Table S5).

After the bioinformatic processing, the results were visualized with Integrative Genomics Viewer (IGV, (Robinson et al., 2011)), plotted for normalized depth of coverage (SVplot), and inspected for the presence of the expected variants to demonstrate orthogonal confirmation. For aneuploidies statistical results based on a depth model have been generated by the Variantyx proprietary intelligence platform. Split/clipped reads alignment and/or SVplot were used for confirmation of structural variants (SVs) that were not fully encompassed inside separate reads due to their size and/or sequence limitations. Short tandem repeat (STR) expansion (Supplementary Table S6) has been analyzed by visual observation of the insertions or clipped reads in the alignments utilizing IGV and by actual repeat count in the expanded reads sequences. In case of very long STR expansion that are not fully spanned by long reads due to their size and/or sequence features, clipped reads were inspected, and maximal observed repeat expansion length has been recorded. Pathogenic STR variants were considered detected and verified when expansion beyond the pathogenic reportable threshold were observed.

The minimal local required coverage threshold was set at 12x for genomic variants and 1,000x for mitochondrial variants. If an expected variant has not been verified and the local observed coverage has been below 12x, an additional sequencing has been performed to increase the depth of coverage when possible. If an expected variant has not been observed with local depth of 12x and above, the variant has been considered false negative. Such approach has been also implemented as a part of the Variantyx’s standard operating procedure that governs the clinical orthogonal confirmation with long reads sequencing of the variants discovered at Variantyx by short reads WGS to ensure high quality of the reported results (Supplementary Table S5).

Analytical performance of the variant detection has been validated in accordance with CLIA guidelines (Centers for Medicare and Medicaid Services, 2023). GIAB samples (NIST, 2023) with available truth sets have been used to create the statistical parameters. Based on the truth set, a randomly selected set of known positive and known negative variants of each variant type were included, and variant detection had been evaluated for each variant type in five separate library preparations and separate sequencing runs, including the runs performed by three different operators on separate days. At least 25 known positive and 25 known negative variants were included for each of the variant types: single nucleotide variants (SNVs), small insertions/deletions under 50 bp, and SVs. NA24385 GIAB (Parikh et al., 2016; Zook et al., 2016; Zook et al., 2020) has been used for analysis of these categories of variants as this sample has the best characterized SV truth set. Positive variants were selected among the variants available in NA24385 while negative variants were selected among variants absent in the NA24385 truth set but present in the NA12878 truth set (for SNV and small insertions/deletions) or previously discovered in other characterized samples (for SV). Analyzed variants were randomly selected in high confidence non-homopolymeric regions. To assure the genome-wide representation of the analyzed variants, the appropriate truth sets were divided into buckets equally spread throughout the genome, and first available variants were selected from each bucket. If bucket selection led to underrepresentation of a chromosome, the first available variants on such chromosomes from the relevant truth set were added to the analyzed lists. Small variants <50bp in mitochondria were evaluated using NA12878 GIAB (Diroma et al., 2014; Zook et al., 2016; Davis et al., 2022) as it has well characterized mitochondrial variants. Positive variants were selected as variants present in NA12878 at ∼100% heteroplasmy while negative variants are known to be absent in NA12878 but were previously discovered in other samples. Due to the relatively low availability of the mitochondrial variants matching the aforementioned criteria, SNVs and small insertions/deletions were combined in a single category, and the whole statistically analyzed set of such variants included 14 positive and 7 negative variants. In the course of analytical validation that included multiple sequencings of the same sample, additional runs were not attempted for variants that were not confirmed in part of the experiments due to low coverage, as the same variants were already addressed in the additional runs, and instead they were excluded from calculations.

Analysis of clinical variant detection has been conducted utilizing samples with characterized pathogenic variants as indicated in the Coriell sample repository (Coriell Institute, 2023) and de-identified clinical samples with pathogenic variants identified in previous testing. For better representation of long reads WGS performance, different types of pathogenic variants were tested in multiple samples when available.

## 3 Results

### 3.1 Analytical validation

To evaluate the general performance of the ONT long reads WGS, Genome in the Bottle (GIAB) samples NA24385 and NA12878 were sequenced with 5 separate library preparations each, and variant verification results have been evaluated against truth sets as described in the Methods according to CLIA test quality guidelines (Centers for Medicare and Medicaid Services, 2023).

Genomic SNVs, small insertions/deletions (and any combinations of thereof) up to 50 bp, and SVs (above 50 bp) were evaluated using NA24385 GIAB truth set for all those types of variants (Table 1), since NA24385 had the most comprehensive truth set for SV (Parikh et al., 2016; Zook et al., 2016; Zook et al., 2020). Small mitochondrial variants <50bp were evaluated using NA12878 GIAB as it has the best characterized mitochondrial variant set (Diroma et al., 2014; Zook et al., 2016; Davis et al., 2022) (Table 1). Assay sensitivity, specificity, positive predictive value (PPV), and accuracy has been calculated separately for each category of variants. All the described types of variants demonstrated high performance characteristics, with sensitivity and specificity >0.950 and >0.970 respectively in every analyzed category. It should be noted that analytical performance of some types of variants was noticeably better than others. For example, sensitivity is higher for SNV detection (0.994) than for small insertions/deletions and for SV (0.976 and 0.958 respectively). The highest sensitivity (1.000) has been achieved for the mitochondrial variants likely due to the high coverage of >1,100x in all tested cases.

**TABLE 1:** Analytical performance validation.

	Metrics	Results
Single Nucleotide Variants (SNV)	True Positives = 164	False Negatives = 1
	False Positives = 0	True Negatives = 168
	Sensitivity	0.99394 (95% CI = 0.9667–0.9998)
	Specificity	1.00000 (95% CI = 0.9783–1.0000)
	PPV	1.00000
	Accuracy	0.99700 (95% CI = 0.9834–0.9999)
Small insertions/deletions * (1–50 bp)	True Positives = 137	False Negatives = 3
	False Positives = 0	True Negatives = 168
	Sensitivity	0.97857 (95% CI = 0.9387–0.9956)
	Specificity	1.00000 (95% CI = 0.9783–1.0000)
	PPV	1.00000
	Accuracy	0.99026 (95% CI = 0.9718–0.9980)
Structural Variants	True Positives = 118	False Negatives = 6
	False Positives = 0	True Negatives = 168
	Sensitivity	0.95833 (95% CI = 0.8977–0.9820)
	Specificity	1.00000 (95% CI = 0.9783–1.0000)
	PPV	1.00000
	Accuracy	0.97945 (95% CI = 0.9558–0.9924)
Mitochondrial Variants ∼ 100% Heteroplasmy	True Positives = 70	False Negatives = 0
	False Positives = 1	True Negatives = 34
	Sensitivity	1.00000 (95% CI = 0.9487–1.0000)
	Specificity	0.97143 (95% CI = 0.8508–0.9993)
	PPV	0.98592
	Accuracy	0.99048 (95% CI = 0.9481 – 0.9998)

^a^ Small insertion/deletions include insertions, deletions, and combinations of thereof below 50bp.

### 3.2 Clinical pathogenic variant analysis

To evaluate the clinical utility of long reads WGS and assess its usability as detection and orthogonal confirmation method for various types of previously discovered pathogenic variants, samples with known clinically relevant variants were sequenced and analyzed as outlined in Methods.

The results are summarized in Table 2 for small variants <50b bp, Table 3 for variants with various levels of mitochondrial heteroplasmy, Table 4 for SVs, and Table 5 for STR expansions which were extensively evaluated as a separate category considering that short reads sequencing cannot always establish the exact number of repeats for longer expansions, while that information might be critical for diagnostic and/or prognostic purposes. Different subcategories of those types of variants were also addressed specifically to demonstrate that long reads WGS-based orthogonal confirmation approach is up to the standards established by the regulatory authorities (New York State. Department Health, 2020).

**TABLE 2:** Clinical validation of pathogenic Small Sequence Changes^a^ (SSC) up to 50 bp detection with long read sequencing.

Sample	Diagnosis	Genotype	Confirmed by ONT Long Reads
NA03251	Mucopolysaccharidosis type IVB Gangliosidosis, generalized GM1, type I galactosidase, beta-1; GLB1	Compound heterozygous Trp273Leu (W273L)/Trp509Cys (W509C) in the GLB1 gene:	Confirmed both variants
		GLB1_c.817_818delTGinsCT | chr3:33,051,979–33,051,980	
		GLB1_c.1527G>T | chr3:3,3051979	
NA01361	Mucopolysaccharidosis type IVA	Compound heterozygous pR61W and pWT405del mutations in the GALNS gene:	Confirmed both variants
		GALNS_c.181C>T | chr16:88842769	
		GALNS_c.1213_1218delTGGACC | chr16:88,824,791–88,824,796	
Sample_1	Convulsions, familial infantile, with paroxysmal choreoathetosis Seizures, benign familial infantile, 2 Episodic kinesigenic dyskinesia 1	PRRT2_c.649dupC (p.Arg217fs) Heterozygous | chr16:29,813,694	Confirmed
Sample_2	Spinocerebellar ataxia 43	MME_c.467delC (p.Pro156fs, rs749320057) Heterozygous | chr3:155,116,689	Confirmed
Sample_3	Intellectual disability, type 39	MYT1L_c.761_764delACAG (p.Asp254fs) Heterozygous	Confirmed
NA11906	Myoclonus epilepsy associated with ragged-red fibers; MERFF transfer RNA, mitochondrial, lysine; MTTK	MTTK, chrM_m.8344A>G	Confirmed
NA11605	Familial optic atrophy; type unknown; Leber-like optic atrophy complex I, subunit ND1; MTND1, mitochondrial	MTND1, chrM_m.3460G>A (Ala52Thr)	Confirmed
Sample_14	Charcot-Marie-Tooth (CMT) disease type 2A2A; Autosomal dominant. Charcot-Marie-Tooth (CMT) disease type 2A2B; Autosomal recessive. Hereditary motor and sensory neuropathy type VIA with optic atrophy; Autosomal dominant	MFN2_c.2219G>C (Trp740Ser)	Confirmed
NA01256	Deficient Alpha-L-Iduronidase; Scheie syndrome	Compound heterozygote: a G>A transition in intron 5, in position −7 from exon 6 (IVS5AS-7G>A) and TGG>TAG at nucleotide 1,293 in exon 9 of the IDUA gene [Trp402Ter (W402X)]	Confirmed

^a^ Small sequence changes (SSC) include Single Nucleotide variants and short insertions, deletions, and combinations of thereof below 50bp.

**TABLE 3:** Clinical validation of Small Sequence Changes^a^ (SSC) up to 50bp with various level of Mitochondrial Heteroplasmy detection with long read sequencing.

Sample	Variant	Known Heteroplasmy level	Concordance ONT Long Reads heteroplasmy level^b^
NA24385	chrM_m.9518C>T	Heteroplasmy = 1%	Concordant at ∼2% Heteroplasmy (20alt/1,274X)
NA24385	chrM_m.13376T>C	Heteroplasmy = 2%	Concordant at ∼3% Heteroplasmy (34alt/1,280X)
NA24385	chrM_m.15737G>A	Heteroplasmy = 2%	Concordant at ∼3% Heteroplasmy (41alt/1,284X)
NA24385	chrM_m.6210T>C	Heteroplasmy = 7%	Concordant at ∼6% Heteroplasmy (82alt/1,201X)
NA11906	chrM_m.8344A>G	Heteroplasmy = 35%	Concordant at ∼39% Heteroplasmy (658alt/1,699X)
NA11605	chrM_m.3460G>A	Heteroplasmy = 95%	Concordant at ∼96% heteroplasmy (1835alt/1905X)
NA24385	chrM_m.1438A>G	Heteroplasmy = 100%	Concordant at ∼96% heteroplasmy (1146alt/1,193X)

^a^ Small sequence changes (SSC) include Single Nucleotide variants and short insertions, deletions, and combinations of thereof below 50bp.

^b^ Observed heteroplasmy is listed as a fraction of alternative variant (alt) out of total depth (X) at the region of interest.

**TABLE 4:** Clinical validation of pathogenic Structural Variant (SV) detection with long read sequencing.

Sample	Diagnosis	Genotype	Confirmed by ONT Long Reads
Sample_4	Chung-Jansen syndrome	67 bp Deletion in 6q14.1q14.1, Heterozygous	Confirmed
NA06047	Miller-Dieker Lissencephaly Syndrome	arr[hg19]17p13.3p13.2(513–5766286)x1	Confirmed
Sample_5	1q21.1 recurrent microdeletion	seq[GRCh38] del(1) (q21.1q21.2) NC_000001.11:g.(146047300_146502700)_(148426300_148816800) del	Confirmed
NA04372	Krabbe disease	chr14q31.3q31.3x1(87,925,162–87,956,828); 33.75 kb	Confirmed
NA05123	Muscular Dystrophy, Duchenne Type; DMD Dystrophin	DMD exon 45–62 duplication; >800,000	Confirmed
Sample_6	Dystrophinopathy Duchenne muscular dystrophy, Becker muscular dystrophy, and DMD associated dilated cardiomyopathy	105.13 kb deletion in Xp21.1p21.1	Confirmed
		Heterozygous	
Sample_7	Klinefelter syndrome and 17q12 deletion syndrome	Klinefelter syndrome 47 XXY	Confirmed both variants
		∼2 Mb Deletion in 17q12q12	
		Heterozygous	
Sample_8	Partial trisomy 19	15.07 Mb Duplication in 19p13.12q12	Confirmed
		Heterozygous	
Sample_9	SATB2-associated syndrome	17.86 Mb Inversion at 2q31.3q33.1	Confirmed
		Heterozygous	
Sample_10	48, XXYY syndrome; Ichthyosis vulgaris, Susceptibility to atopic dermatitis	48, XXYY syndrome	Confirmed
Sample_11	Recurrent 16p11.2 microdeletion syndrome	Heterozygous, pathogenic deletion of approximately 322.5 kb within the distal region of chromosome 16p11.2	Confirmed
Sample_13	Leri-Weill dyschondrosteosis	∼1.9 Mb deletion in Xp22.33p22.33	Confirmed
	Short stature; Langer mesomelic dysplasia	Heterozygous	
Sample_15	Mulchandani–Bhoj–Conlin syndrome	Maternal UPD of chromosome 20 (UPD(20)mat)	Confirmed
NA11601	Neurofibromatosis, type I; NF1	Insertion of an Alu element close to exon 6 of NF1	Confirmed
NA13805	Ataxia-telangiectasia; ATM	ATM_c.935_936insAlu (p.Leu312fs)	Confirmed

**TABLE 5:** Clinical validation of Short Tandem Repeats (STR) detection with long read sequencing.

Sample	Diagnosis	Genotype (Gene and Repeat count)	Reportable repeat count threshold	Confirmed by ONT Long Reads
NA23709	Spinal and Bulbar Muscular Atrophy, X-Linked 1; SMAX1	AR, 51 repeats (male, 1 copy of chrX)	35	Confirmed
NA13716	Dentatorubral-Pallidoluysian Atrophy; DRPLA	ATN1, 16/68 repeats	36	Confirmed
NA13515	Huntington Disease; HD	HTT, 16/66 repeats	36	Confirmed
NA13503	Huntington Disease; HD	HTT, 17/45 repeats	36	Confirmed
NA03561	Spinocerebellar Ataxia 7; SCA7	ATXN7, 8/62 repeats	27	Confirmed
NA06926	Spinocerebellar AtaxiaA 1; SCA1; ATAXIN 1; ATX1	ATXN1, 29/52 repeats	36	Confirmed
NA06151	Machado-Joseph Disease; MJD; ATAXIN 3; ATXN3	ATXN3, 24/74 repeats	27	Confirmed
Sample_12	Frontotemporal dementia and/or amyotrophic lateral sclerosis	C9ORF72, 1 expanded allele	25	Confirmed
NA15850	Friedreich ataxia 1	FXN, 650/1,030 repeats	60	Confirmed
NA16213	Friedreich ataxia 1 (Carrier, not affected)	FXN, 1 normal allele/420 repeats	60	Confirmed
NA05164	Dystrophia myotonica 1	DMPK, 21/340 repeats	36	Confirmed
NA23378	Dystrophia myotonica 1	DMPK, 1 normal allele/∼80–90 repeats	36	Confirmed
NA03759	Dystrophia myotonica 1	DMPK, 1 normal allele/2000 repeats	36	Confirmed
NA06897	Fragile X Syndrome	FMR1, 477 repeats (male, 1 copy of chrX)	45	Confirmed
NA07175	Clinically Normal	FMR1, 30/23 repeats (female, 2 copies of chrX)	45	Confirmed
NA09237	Fragile X Syndrome	FMR1, 931–940 repeats (male, 1 copy of chrX)	45	Confirmed
NA20239	Fragile X Syndrome (Carrier, not affected)	FMR1, 20/183–193 repeats (female, 2 copies of chrX)	45	Confirmed

### 3.3 Small sequence variants

**FIGURE 1:**
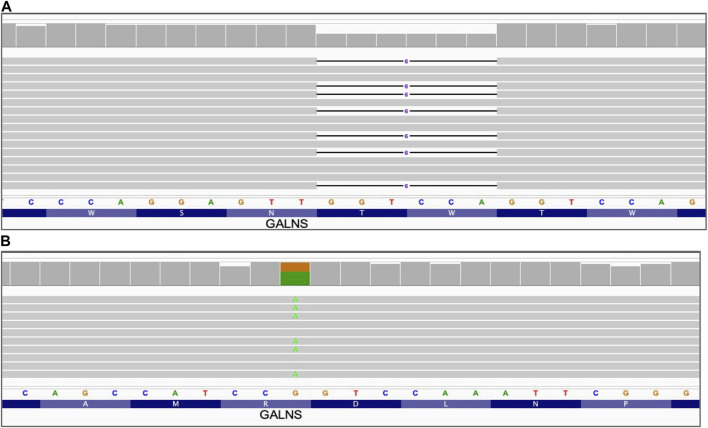
Small sequence changes. Alignment view of the long read WGS of sample NA01361, GALNS gene. Each thick gray horizontal line represents a separate read. Narrow horizontal lines with numbers represent deletions of the indicated length (in base pairs). Top of each panel represents a local coverage depth and color coded when applicable to indicate nucleotide variants at the corresponding position. String of letters below the panels indicate reference nucleotide sequence, and colored nucleotides over the read sequences stand for the alternative allelic variant. **(A)** Deletion of 6 nucleotides GALNS_c.1213_1218delTGGACC; **(B)** Single nucleotide variant GALNS_c.181C>T.

**FIGURE 2:**
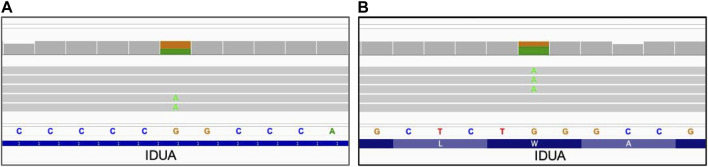
Phasing of compound heterozygous small sequence changes. Alignment view of the long read WGS of sample NA01256 with two variants in the IDUA gene. Each thick gray horizontal line represents a separate read. Top of each panel represents a local coverage depth and color coded when applicable to indicate nucleotide variability at the corresponding position. Panels A and B are continuation of each other with preserved order of reads spanning both of them. Indicated variants are located within the distance spanned by those reads and can be reliably phased, confirming association with alternative haplotypes. String of letters below the panels indicate reference nucleotide sequence, and colored nucleotides over the read sequences stand for the alternative allelic variants. **(A)** Single nucleotide variant IDUA_c.590-7G>A; **(B)** Single nucleotide variant IDUA_c.1205G>A.

Analysis of the long reads WGS results of samples with known SNVs and small insertions/deletions below 50bp has demonstrated that this approach combined with visual variant inspection can reliably verify the presence of such variants (Table 2; Figures 1, 2). Moreover, long reads WGS sequencing provides an additional benefit of phasing variants, which might be a critical piece of clinically relevant information in case of recessive pathogenic variants, as compound heterozygous renders the patient with two affected copies of the gene. Figure 2 demonstrates this capabilit y in a sample carrying a compound heterozygous combination of two *IDUA* variants separated by 1,075 bp and clearly attributable in long reads sequencing results to separate alleles. That distance is easily covered by long reads, leading to a reliable phasing, while short reads have a limited phasing capacity and in such cases might require trio familial sequencing. Moreover, long reads WGS provides reliable variant phasing throughout the entire length of the majority of clinically relevant genes.

### 3.4 Mitochondrial heteroplasmy

**FIGURE 3:**
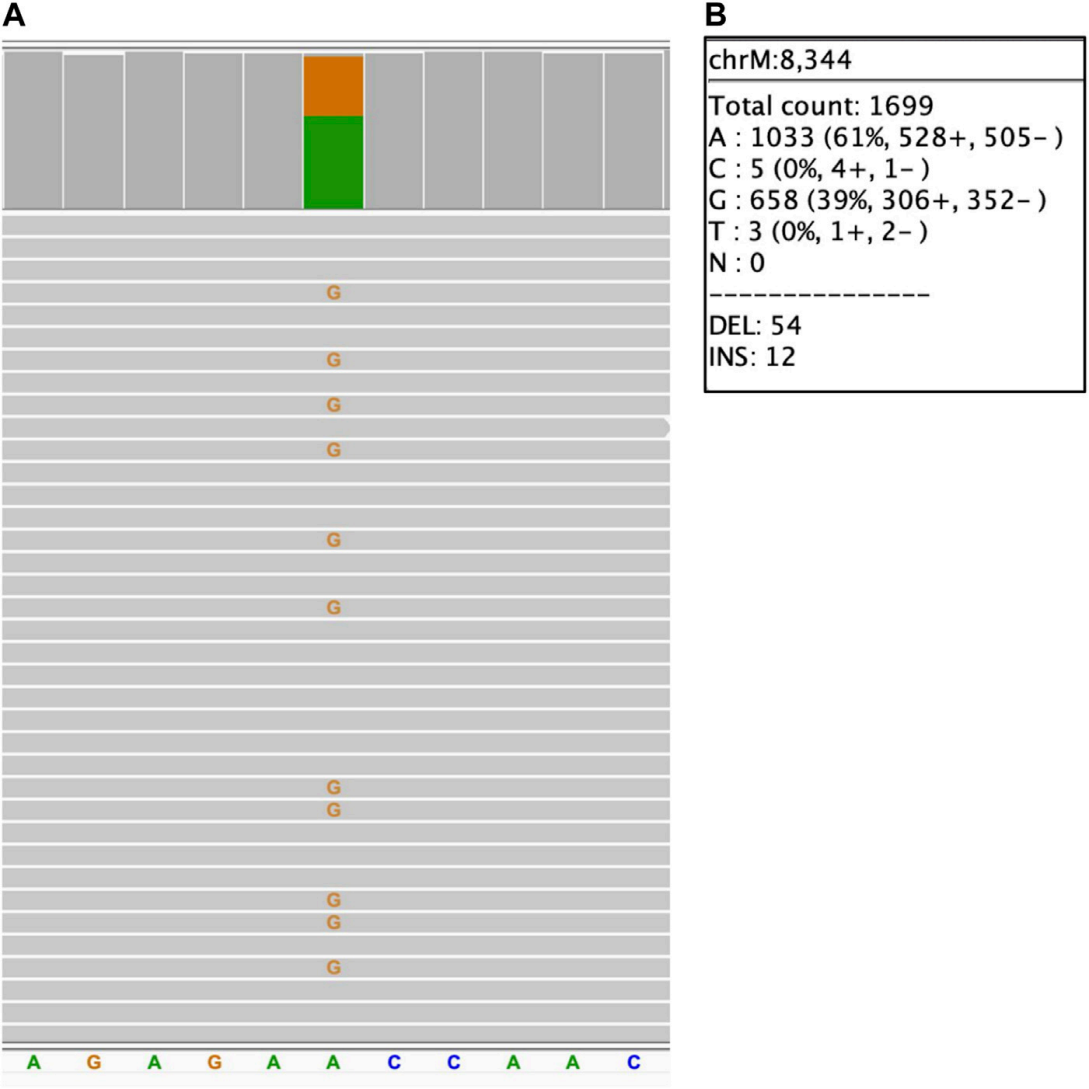
Mitochondrial small sequence change with heteroplasmy. NA11906 sample. **(A)** MTTK_chrM_m.8344A>G single nucleotide variant, alignment view of the long read WGS. Each thick gray horizontal line represents a separate read. Top of each panel represents a local coverage depth and color coded when applicable to indicate nucleotide variability at the corresponding position. String of letters below the panel indicate reference nucleotide sequence, and colored nucleotides over the read sequences stand for the alternative allelic variants. Due to the very high depth of coverage for the mitochondrial variants (>1,000x), the image shows only a small fraction of reads; **(B)** MTTK_chrM_m.8344A>G heteroplasmy level detected with long reads WGS.

For mitochondrial variants, the level of heteroplasmy that might significantly affect the variant impact (Wallace and Chalkia, 2013; Laricchia et al., 2022), is of high clinical importance. To assess the limit of detection for mitochondrial heteroplasmy, the concordance of the heteroplasmy level to the available information has been evaluated in previously characterized samples acquired from Coriell repository. The results are summarized in Table 3, demonstrating that long reads WGS allows detection of the heteroplasmy level close to the expected level: an approximate concordance of the level of heteroplasmy (with not more than 1% difference at heteroplasmy expected levels below 30% and not more than 4% difference at the heteroplasmy levels above 30%) has been observed. Figure 3 demonstrates a 39% heteroplasmy detected for a known pathogenic variant in MTTK gene in NA11906 sample with expected heteroplasmy level around 35% (Coriell Institute, 2023; MRC Holland, 2023).

### 3.5 Short tandem repeats (STRs)

Expansions of STRs are a known cause of multiple genetic disorders, and due to the high instability of such STRs they have normal and pathogenic ranges, and typically additional categories such as intermediate, permutation, *etc.* (Willems et al., 2014; Depienne, 2021; Depienne and Mandel, 2021). A difference between normal and abnormal range might be only a single or just a few repeats, and even for repeats well within pathogenic expansion range, exact length and presence of interrupting repeats might significantly affect the disease onset and severity (Weisman-Shomer et al., 2000; Chintalaphani et al., 2021).

**FIGURE 4:**
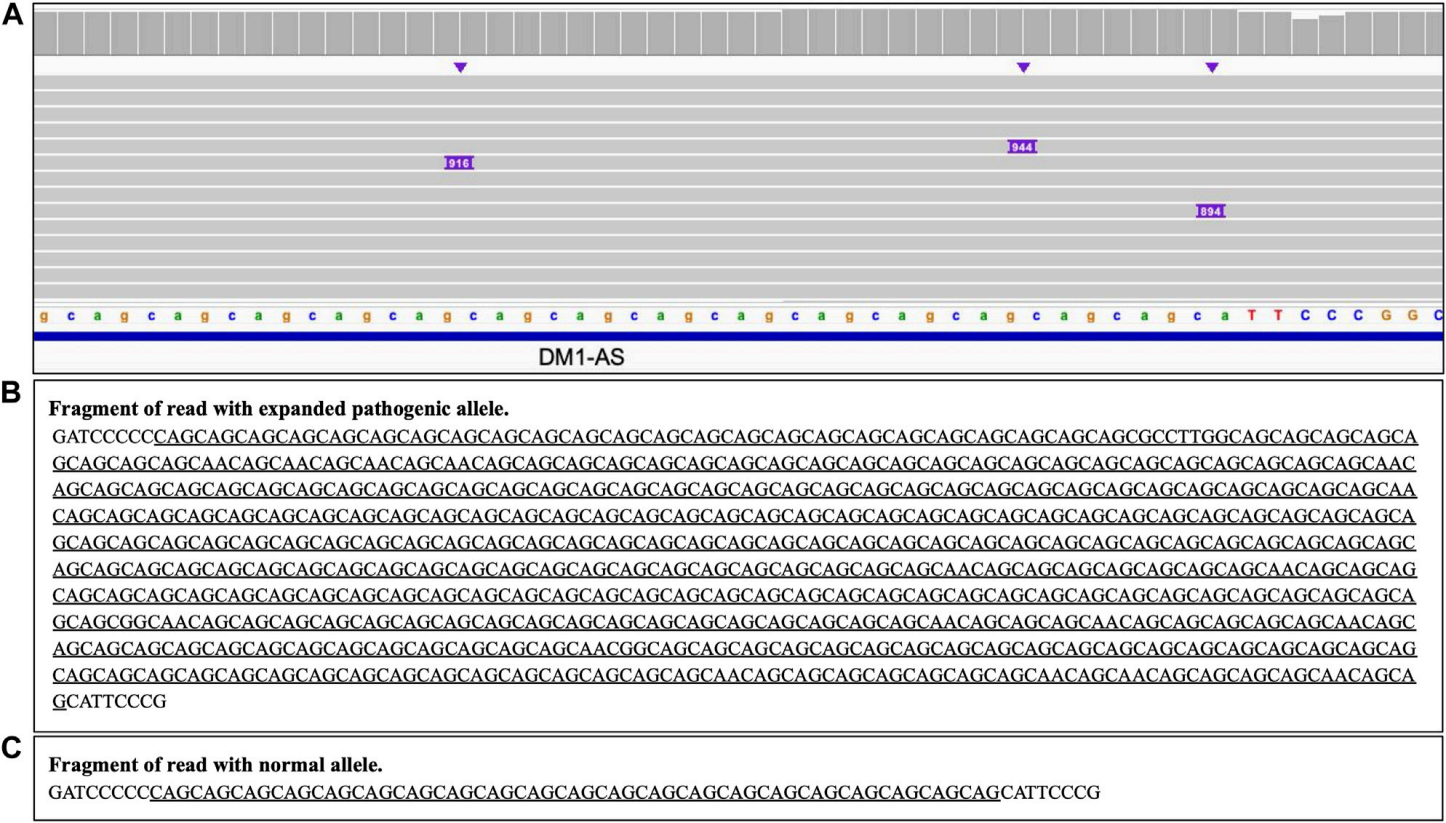
DMPK heterozygous STR expansion. Long reads WGS sequencing of sample NA05164 (repeat count 21/340 per Coriell repository information). **(A)** Alignment view of the STR region. Each thick gray horizontal line represents a separate read, with purple rectangles with numbers indicating insertions of the corresponding length (in bp). Top of the panel represents a local coverage depth. Purple triangles above the reads indicate the locations of the insertions. Reads with long insertion correspond to a pathogenic expanded repeat; **(B, C)** Examples of the sequence of the reads around the STR region (underlined): expanded pathogenic allele (313 CAG +21 interrupting repeats, panel **(B)** and normal allele (panel C).

**FIGURE 5:**
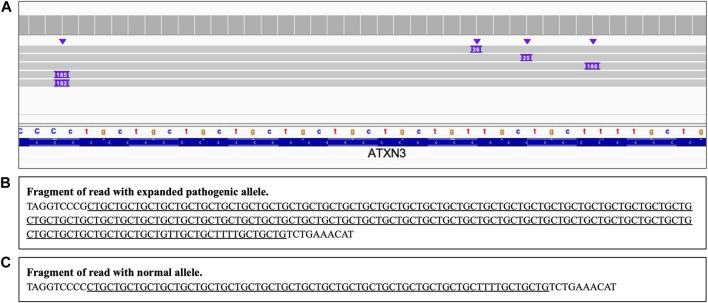
ATXN3 STR heterozygous STR expansion. Long reads WGS sequencing of sample NA06151 (repeat count 24/74 per Coriell repository). **(A)** Alignment view of the STR region. Each thick gray horizontal line represents a separate read, with purple rectangles with numbers indicating insertions of the corresponding length (in base pairs). Top of the panel represents a local coverage depth. Purple triangles above the reads indicate the locations of the insertions. Reads with >180 bp insertion correspond to a pathogenic expanded repeat, while reads with short ∼25 insertion correspond to an allele within the normal repeat range; **(B, C)** Examples of the sequence of the reads around the STR region (underlined): expanded pathogenic allele (74 CTG +3 interrupting repeats, panel **(B)** and normal allele (panel C).

Analysis of the long reads WGS of the true positive samples with STRs of various lengths (Table 5) has demonstrated that this approach can be reliably used for STR expansion detection. Aligned sequencing results were inspected with IGV viewer, in which the repeat expansions are visualized as insertions of the appropriate length, and sequence of the relevant reads had been examined to verify the STR expansions and examine the exact repeats count and the presence of interruptions. Examples of STR expansion in DMPK and in ATXN3 repeat regions are displayed in Figures 4, 5 respectively. Each alignment demonstrates two types of reads: one corresponding to a normal allele and one with long insertion corresponding to a pathogenic expanded allele. Sequences of STR regions in those two types of reads are displayed under each alignment, confirming the expanded repeat count. Notably, despite the relatively low depth (∼12x) of the performed long reads WGS, it has not only confirmed the presence of the expected pathogenic repeat expansions, but also has identified the existence of mosaic mixes of the expansions of different length in the same patient due to somatic variability, as it has been published previously (Chiu et al., 2021). In addition, this approach also demonstrated an ability to detect the presence of the interrupting repeats, which are considered a useful diagnostic and prognostic feature (Weisman-Shomer et al., 2000; Chintalaphani et al., 2021). Considering that for many larger STR expansions testing based on short reads NGS and some other techniques can only approximately establish the expansion range and do not address the location of interrupting repeats and somatic variability in length, some discrepancies between results obtained by long reads sequencing and other methods might be expected. Nevertheless, in all the examined cases the expected pathogenic or normal range of the STR expansions has been confirmed with long reads WGS, which supports the validity of such approach for genetic testing as well as orthogonal verification of the results of other previous tests.

### 3.6 Structural variants (SVs)

Depending on the length and specific features of the SV, long read WGS results might either allow a direct IGV visualization of the whole variant, or additional ways of review of the data might be required. SVs disrupt the linear flow of the genomic sequence and might be reflected in the sequencing results as soft clipped/split reads mapped partially at the breakpoint coordinates at both ends of the variants. Such split/clipped signal is visualized in IGV. In addition, copy number variants (CNVs) lead to a change in depth of coverage that might be visualized with depth-based SVplot, although that feature performs best for longer variants (∼ above 300 kb). Aneuploidies are a subcategory of CNV, and Variantyx proprietary Intelligence platform allows to inspect such variants directly with numerical output reflecting read coverage distribution over the genome. Therefore, different strategies were applied for verification of structural variants depending on their type and length.

#### 3.6.1 Mobile element insertions (MEI)

**FIGURE 6:**
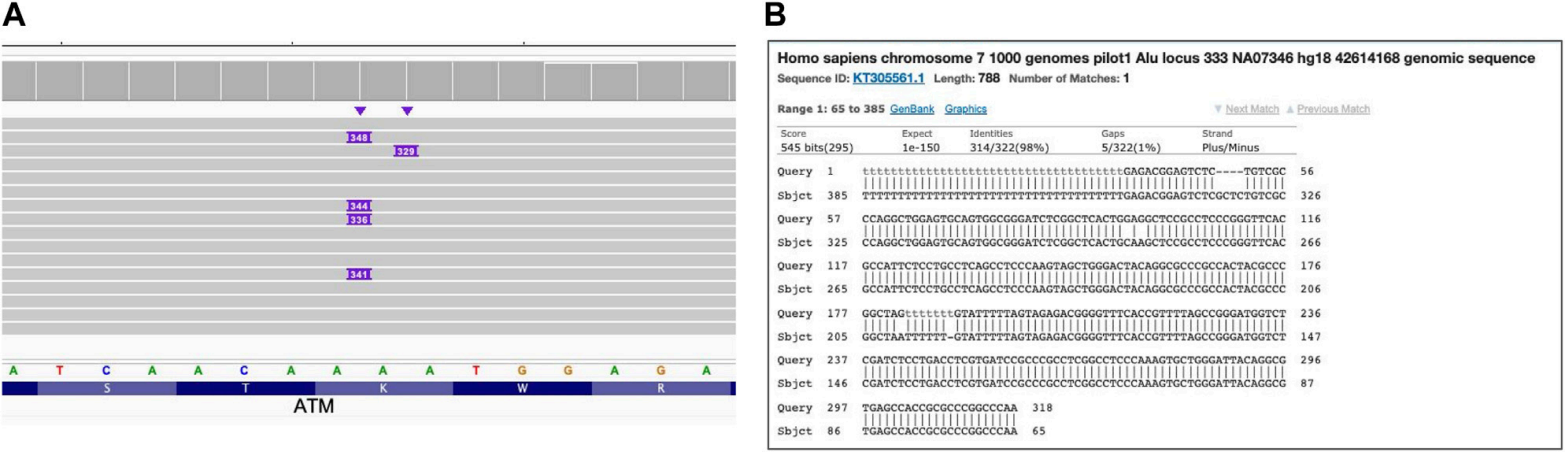
Alu mobile element insertion in ATM gene. Long read WGS of sample NA13805. **(A)** Alignment view Each thick gray horizontal line represents a separate read, with purple rectangles with numbers indicating insertions of the corresponding length (in base pairs). Top of the panel represents a local coverage depth. Purple triangles above the reads indicate the locations of the insertion; **(B)** blast (https://blast.ncbi.nlm.nih.gov/Blast.cgi) results verifying Alu mobile element sequence of the insertion.

Mobile elements now comprise a large portion of the human genome and are still duplicating, generating new SVs that might be clinically relevant (Stewart et al., 2011; Niu et al., 2022). Figure 6 demonstrates the results of long read WGS of Alu MEI insertion in the ATM gene. The insertion size (∼300bp) is clearly visible with IGV as an insertion and compatible with expected Alu length (Pray L. A., 2008) and the Alu sequence confirmed by a blastn search (BLAST, 2023).

#### 3.6.2 Inversions

**FIGURE 7:**
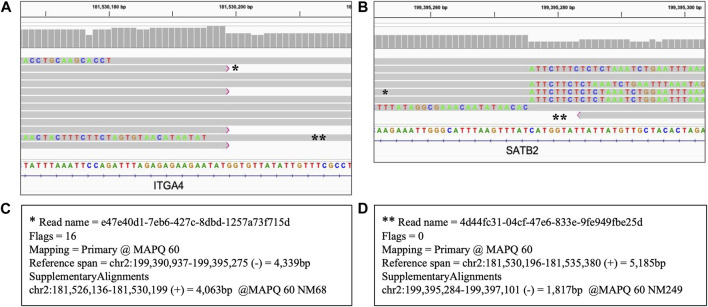
Heterozygous inversion of 17.86 Mb seq[GRCh38] inv(2) (q31.3q33.1). Long read WGS of Sample_9. Each thick gray horizontal line represents a separate read. String of letters below the panels indicate reference nucleotide sequence, and colored nucleotides over the read sequences stand for the nucleotides not matching the reference; strings of mismatches of that kind reflect the regions that got disjoined from the original reference location. Panels A and B represent two ends (breakpoint) of the same variant with the same reads partially mapping to the reference sequence at both ends separated by distance and orientation (i.e., split reads). Two examples of such split reads are marked with * and ** respectively. Panels C and D display primary and supplementary alignments for different reads, i.e., detached parts of the same read mapping with the best score and the second best score respectively to the different positions. **(A)** Left breakpoint alignment view; **(B)** Right breakpoint alignment view; **(C)** Alignment details of the reads marked with * in panels **(A)** and **(B)**. That read is mapped partially as a primary alignment at the left breakpoint coordinate and partially as a supplementary alignment in opposite orientation at the right breakpoint coordinate; **(D)** Alignment details of the read marked with ** in panels **(A)** and **(B)**. That read is mapped partially as primary alignment at the right breakpoint coordinate and partially as a supplementary alignment in opposite orientation at the left breakpoint coordinate.

Inversions are expected to be reflected by sequencing reads with one part mapped to the reference at one breakpoint of the inversion and another part of the same read mapped to another breakpoint in an opposite direction (NCBI, 2023). Figure 7 shows the results of the long read WGS of 17.86 Mb inversion displaying those features. Split reads are present at both end coordinates of the inverted fragment, mapping as expected in direct orientation at one end and in the reversed orientation at another end. Therefore, though that SV is too long to be encompassed by the 10 kb long reads used in our assay, that assay still allows a reliable identification as the results are consistent with both the coordinates and the mapping pattern of the inversion.

#### 3.6.3 Copy number variants (CNVs)

**FIGURE 8:**
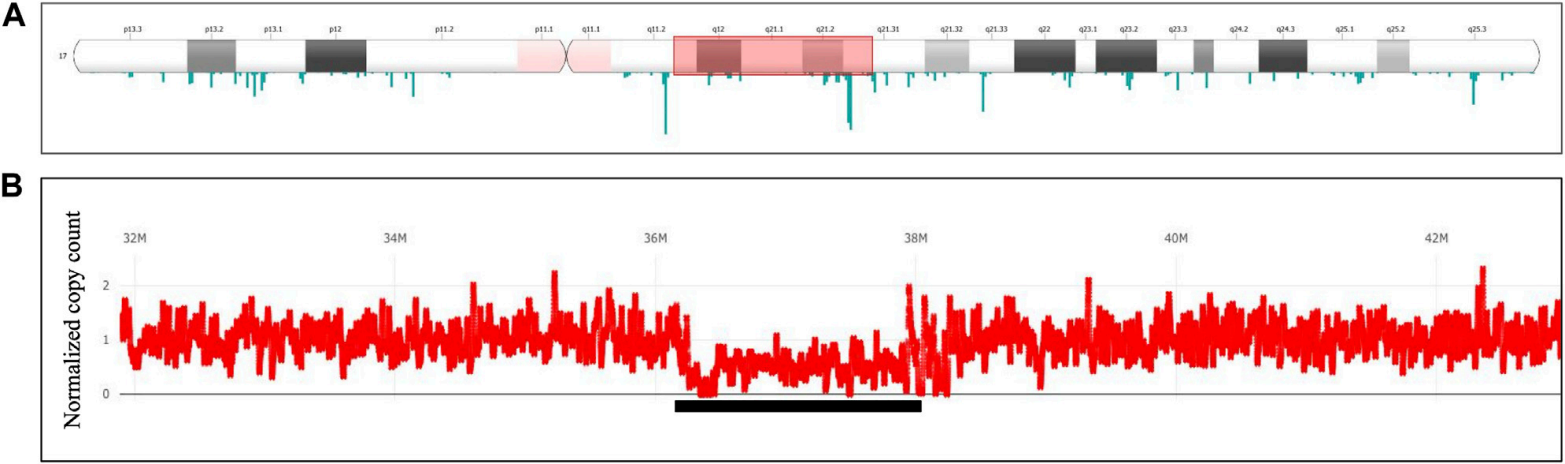
Heterozygous deletion of ∼∼2 Mb Deletion in 17q12q12. Long read WGS of Sample_7. **(A)** Ideogram depicting chromosome 17. The whole length of chromosome 17 with both arms (p, q) and a centromere is shown, including the banding pattern. Shaded fragment around q12-q21.2 indicates region of interest that is magnified in panel **(B)** Depth of coverage distribution over the selected fragment. Axis Y indicates normalized copy counts, with average count 1 equivalent to normal 2 copies of the chromosome. Underlined is a region with average copy count drop to ∼0.5 hence representing heterozygous deletion.

For verification of CNVs, depth-based analysis has been utilized. Figure 8 shows the results of the long read WGS sequencing of ∼2.0 Mb heterozygous deletion visualized with the depth-based SVplot. Graph at Figure 8B represents depth distribution expressed as normalized copy count where value 1 corresponds to 2 copies of chromosome. Drop of the normalized copy count to ∼0.5 over the region of interest confirms a presence of the heterozygous deletion.

#### 3.6.4 Chromosomal aneuploidies

Variantyx proprietary intelligence platform allows detection of aneuploidies based on the distribution of sequencing reads per chromosome normalized by number of reads per genome. Table 6 demonstrates long reads WGS results of a case with 48, XXYY syndrome. Numerical values reflecting normalized ploidy were generated based on the internal model. Two copies of chromosome X and two copies of chromosome Y can be observed hence confirming the 48, XXYY genotype. Examples of normalized ploidy in female and male samples without aneuploidies are shown for comparison in Supplementary Table S2, 3 respectively.

**TABLE 6:** Aneuploidy confirmation for 48, XXYY syndrome (Sample_10).

Chromosome	Predicted Ploidy	Normalized Ploidy	Read Coverage
chr1	2	2.01	10.65
chr2	2	1.99	10.62
chr3	2	2.0	10.68
chr4	2	1.98	10.56
chr5	2	1.99	10.6
chr6	2	2.0	10.64
chr7	2	2.0	10.63
chr8	2	1.99	10.59
chr9	2	2.0	10.7
chr10	2	2.02	10.72
chr11	2	2.0	10.62
chr12	2	2.0	10.64
chr13	2	1.98	10.56
chr14	2	1.98	10.54
chr15	2	2.01	10.68
chr16	2	2.03	10.69
chr17	2	2.03	10.69
chr18	2	1.99	10.61
chr19	2	2.05	10.71
chr20	2	2.0	10.7
chr21	2	1.99	10.68
chr22	2	2.0	10.66
chrX	2	1.97*	10.52
chrY	2	1.91*	10.45

^a^ Normalized ploidy ∼2x for both X and Y chromosomes.

#### 3.6.5 Uniparental disomy (UPD)

UPD occurs when a person inherits two copies of a chromosome, or part of a chromosome, from one parent and none from another one.

Confirmation of UPD variants requires inspection of small sequence changes in the target region that are homozygous alternate in one parent, while reference in another one. To be informative for UPD verification, homozygous variants should be present in the parent that did not pass any alleles to the proband, and the reference parent should be the one that served as a source of UPD. Absence of those variants in the proband is a confirmation that indeed both copies of the region of interest were inherited from the parent with reference variants. It should be noted that such an approach could be applied ubiquitously for confirmation of UPD of any type (isodisomy or heterodisomy). Considering that such UPD analysis requires parental genetic information, it should be used either in trio sequencing or with reuse of the parental info from previous testing.

Historically, traditional assays use at least two informative loci to confirm UPD (del Gaudio et al., 2020). For improved reliability of the confirmatory UPD analysis, Variantyx has elected to establish a standard operation procedure to test at least 20 such loci randomly spread throughout the UPD region(s) to provide statistically significant results for confirmation (95% confidence interval). Verification of depth of coverage is also performed with variant review to rule out a deletion in the region. Since UPD analysis relies on small sequence changes, it inherits the performance characteristics of those variants and analytical approach to their inspection in long reads WGS.

**FIGURE 9:**
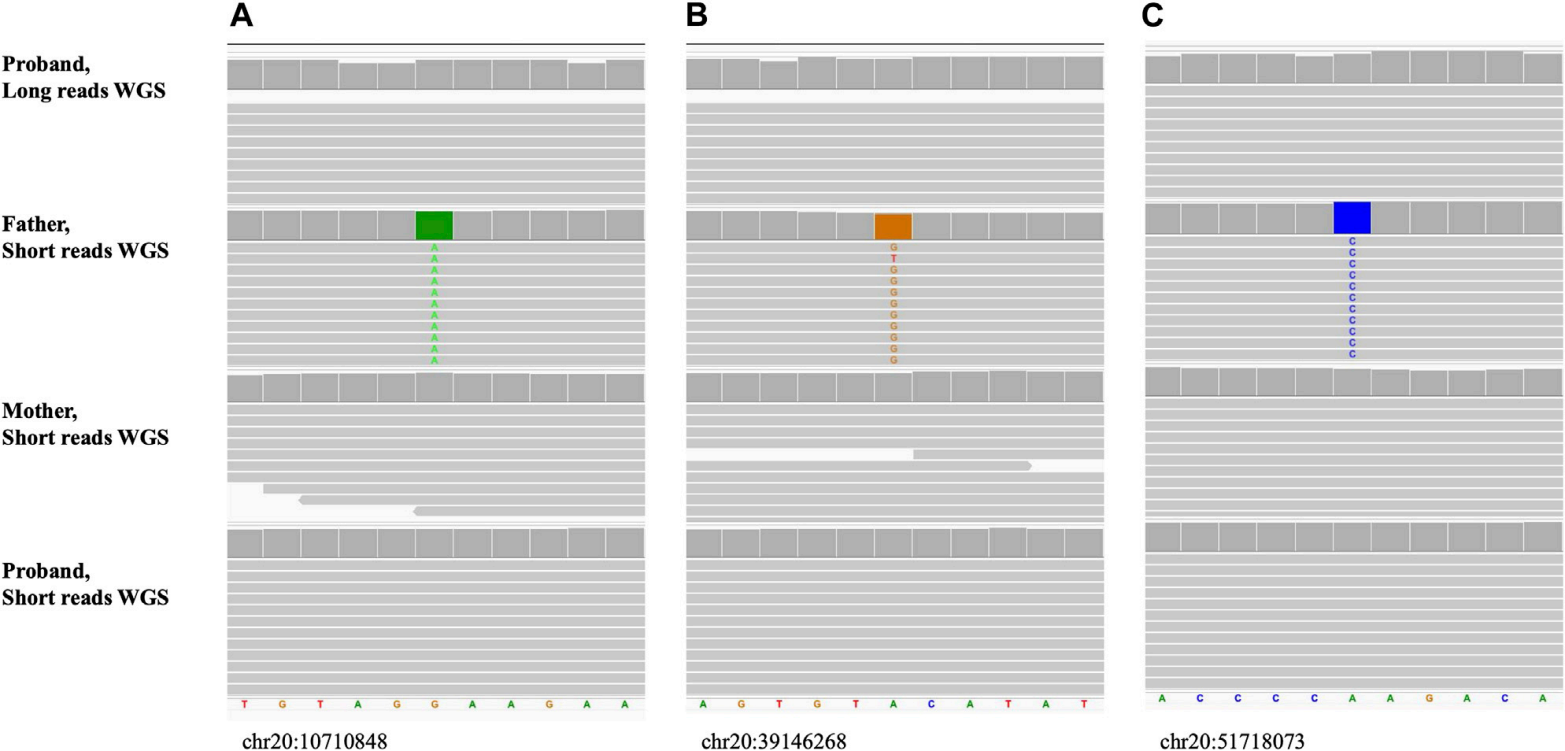
Maternal uniparental disomy, UPD(20)mat (Sample_15). The alignment view of the confirmatory long reads WGS in proband and original short reads familial trio WGS. Order top to bottom: long reads confirmatory WGS in proband, short reads initial WGS in father, mother, proband. Each thick gray horizontal line represents a separate read. String of letters below the panels indicate reference nucleotide sequence, and colored nucleotides over the read sequences stand for the nucleotides not matching the reference. **(A–C)** Examples of small sequence changes used for UPD confirmation. Variants are homozygous alternate in father and reference in mother. Coordinates of the variants on hg38 are shown under each alignment.

Figure 9; Supplementary Figure S1 show the long read WGS results of a maternal UPD case (UPD(20)mat) for which informative loci were selected to be a reference in the mother and homozygous alternate in the father, based on the prior short-read trio sequencing results. The proband was a reference for all 20 of the inspected variants confirming maternal only inheritance of both alleles.

## 4 Discussion

Incorporation of long read WGS technology expands the genetic testing capabilities further beyond the limits set by the use of short read NGS. As we have demonstrated, long reads based WGS can be used to simultaneously detect multiple types of variants, including repeat expansions, mobile element insertions, structural variants, uniparental disomy, deletions/duplications, small sequence changes, and mitochondrial variants. It also allows collection of additional clinically relevant information that would not be otherwise available, such as precise STR expansions size and sequence, phasing of the potentially compound variants, *etc.*

Consolidating current standard of care multi-platform genetic tests into one unified test with comprehensive reporting is highly desirable in the clinical genomics space to improve diagnostic yields, shorten test turnaround times and maximize window of opportunity for clinical management and intervention.

Despite the potential of the ONT long read WGS genetic testing, it still has its limitations that do not allow the use of the current commercially available technology generation as a standalone approach for some types of variants. One of the main limitations is a low raw read accuracy provided by the currently released flow cell (R9.4) and chemistry versions, translated, for example, into a suboptimal accuracy of detection of small insertions/deletions/combinations (Oxford Nanopore Technologies, 2023d.; Marx, 2023). Of course, this limitation is mostly apparent at low depth of sequencing, and can be compensated by increased coverage, which, however, subsequently makes the whole process more expensive and cost prohibitive for broad clinical use. Nevertheless, with the recent introduction of a new version of flow cells and chemistry, constituting Q20+ ONT chemistry (Oxford Nanopore Technologies, 2023e), this disadvantage might soon be resolved, although for now the most advanced ONT chemistry is available as early access only, susceptible to frequent adjustments and hence not well suitable for clinical use. At current time, Variantyx has adapted a workaround solution for that issue that includes a visual inspection of each confirmatory result which allows to increase the reliability of the variant detection even with the known limitation of low depth coverage. Presently at Variantyx the ONT long reads sequencing is utilized only for confirmatory orthogonal verification of the variants detected with Illumina based short read PCR-free WGS, hence following the same procedure that has been implemented for validation: chromosomal coordinates of the detected variants are used for inspection of the long reads. Such approach is enabled by the Variantyx proprietary diagnostic console allowing coordinate-based easy visualized access to the appropriate locations in the long reads, as well as inspection of the normalized plotted depth and of the visual STR accession aid when applicable. Direct automated primary detection of variants with low pass ONT long reads is not feasible at the current moment, though should become possible with development of improved variant detection bioinformatic tools and population models coupled with improved sequencing quality. While those advanced options have not become available yet, we choose to maintain a careful inspection of all reportable variants. We believe that responsible use of any innovative technologies including ONT long reads should err on the side of caution, investing more effort rather than risking any mistakes that might have a detrimental effect on patients’ health.

In general, longer reads of long read WGS are expected to provide better resolution of genomic regions that are not uniquely mappable in short read based WGS, such as pseudogenes or highly variable genes. Unfortunately, as differentiation of such highly similar regions often relies on very limited number of sequence variations, a relatively low accuracy of the current ONT long reads basecalling makes it not well suitable for such applications. Wide introduction of the Q20+ generation of the ONT chemistry that has not yet been fully released by the manufacturer is expected to improve mapping resolution. An alternative way to resolve this disadvantage, when only limited regions are of interest, is a targeted sequencing that can provide an increased depth of coverage and hence increased accuracy of select regions without being cost prohibitive. Conveniently, targeted sequencing is available for ONT technology not only at the library preparation level, but also as adaptive sampling at *in silico* level, hence allowing for more flexibility (Payne et al., 2021; Stevanovski et al., 2022).

Despite the current aforementioned limitations of the ONT sequencing, it, being a long read-based technology, provides a significant advantage in the detection of some types of variants. Many structural variants are detectable by short reads sequencing only due to depth changes and discordant mapping of the paired reads, which leads to a significant reduction in sensitivity of detection of smaller SVs, particularly below 1,000 bp (Neerman et al., 2019). Such smaller SVs though can be fully encompassed by long reads, or at least reliably anchored at each end, hence greatly improving their detection rate and allowing the precise identification of the positions of the breakpoints and specific length/sequence. We have demonstrated such capability of long reads for inversions, deletions, insertions including mobile elements, and STR expansions, as well as phasing of compound genetic variants.

For large STR expansion, allele length and sequence discovered with a long read-based approach is in some cases more precise than the approximate results generated by a variety of other assays. Short reads NGS has a limited power to detect the length of long pathogenic STR expansions due to the intrinsic limitation of the read size (Fang et al., 2022) and hence forced to rely on statistical approach, Southern blot addresses only the fragment length but not the sequence, and repeat-primed PCR has intrinsic limitations due to possible stutter (Chintalaphani et al., 2021). In addition, long reads WGS allows to resolve the haplotypes of the expanded alleles, which is critical particularly for interpretation of recessive pathogenic STR expansions such as FXN and RFC1.

We have demonstrated the ability of long reads WGS to successfully identify pathogenic STR allele expansions in all analyzed cases. In addition, even in the low depth (12x) long reads WGS allows to identify the presence of somatic variability (Chiu et al., 2021) and location of interrupting sequences in the STR expansions (Weisman-Shomer et al., 2000). The presence and location of such interruptions in the expanded STRs along with the expansion length might be of utmost importance for diagnostic and prognostic purposes (Weisman-Shomer et al., 2000; Chintalaphani et al., 2021). Such information, together with epigenetic modification information that is also available in the ONT long reads, presents a significant advantage for clinical genetic testing.

Overall, long reads WGS utilizing ONT chemistry currently released for clinical use is not ideal for a standalone comprehensive clinical genetic testing. However, when it is used to complement short read based WGS, it greatly enhances capabilities of the combined assay and also provides a reliable system for intrinsic orthogonal verification of the detected genetic variants. Based on this validation study, we have implemented the ONT long read-based WGS as a supplementary technology for routine clinical genetic testing.

With further development of the ONT technology to improve basecalling and significantly reduce sequencing costs it has a potential to make short read based NGS obsolete and to become a basis of a comprehensive first line diagnostic test every patient with suspected genetic disease would benefit from.

## Data Availability

The datasets presented in this study can be found in online repositories. The names of the repository/repositories and accession number(s) can be found below: Sequencing data can be found in Sequence Read Archive (SRA) NCBI under bioproject accession PRJNA930347.
