# Supplementary material for: ONT long-read WGS for variant discovery and orthogonal confirmation of short read WGS derived genetic variants in clinical genetic testing
Source: Front Genet. 2023 Apr 21;14:1145285. doi: 10.3389/fgene.2023.1145285 (PMC10160624; doi:10.3389/fgene.2023.1145285)
Supplement: Supplementary file 1 [file DataSheet1.pdf]

Supplementary Material

ONT long read sequencing for variant discovery and orthogonal confirmation of short read WGS derived genetic variants in clinical genetic testing

Ludmila Kaplun[1]*, Greice Krautz-Peterson[1], Nir Neerman[1], Christine Stanley[1], Shane Hussey[1], Margo Folwick[1], Ava McGarry[1], Shirel Weiss[1], Alexander Kaplun[1].

[1]Variantyx Inc, Framingham, MA, USA

*** Correspondence:** Ludmila Kaplun: lucy.kaplun@variantyx.com

1 Supplementary Data

Supplementary Figure 1. Maternal uniparental disomy, UPD(20)mat (Sample_15).
The alignment view of the confirmatory long reads WGS in proband and original short reads familial trio WGS. Order top to bottom: long reads confirmatory WGS in proband, short reads initial WGS in father, mother, proband. Each thick gray horizontal line represents a separate read. Narrow horizontal lines with numbers represent deletions of the indicated length (in base pairs). Top of each panel represents a local coverage depth and color coded when applicable to indicate nucleotide variants at the corresponding position. **(A-T)** All twenty small sequence changes used for UPD confirmation. Variants are homozygous alternate in father and reference in mother. Coordinates of the variants on hg38 are shown under each alignment.

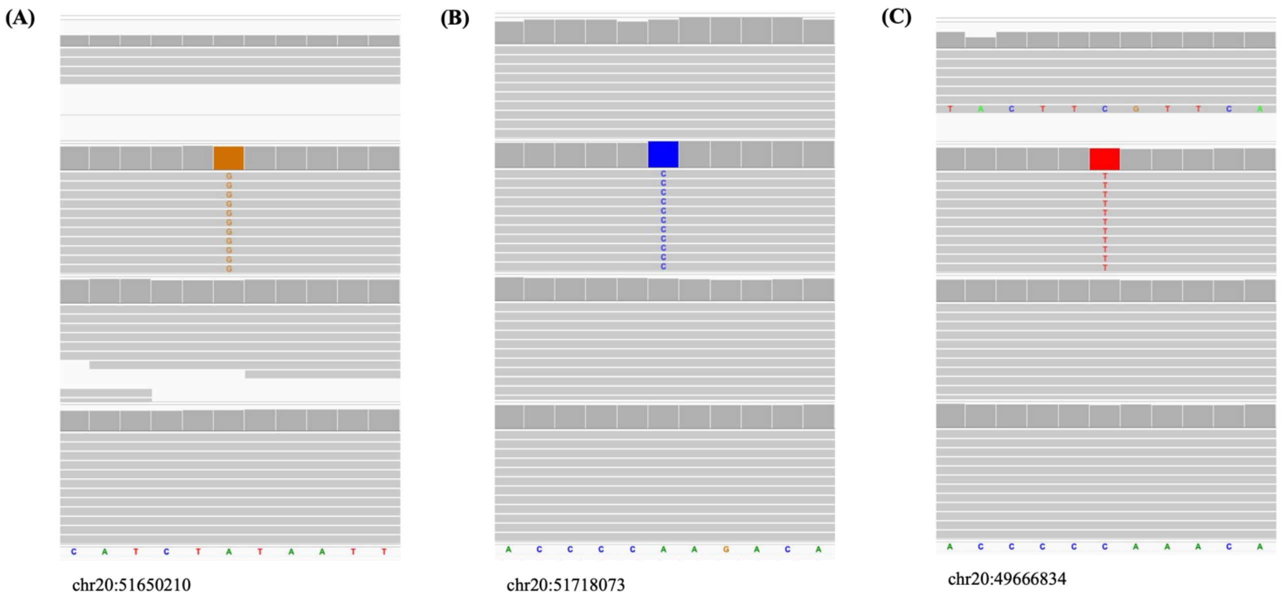

(D)

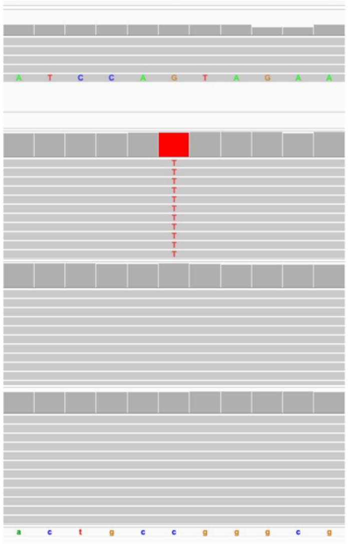

chr20:50047265

(E)

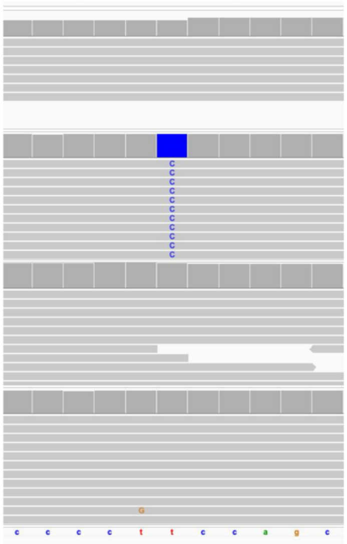

chr20:51252568

(F)

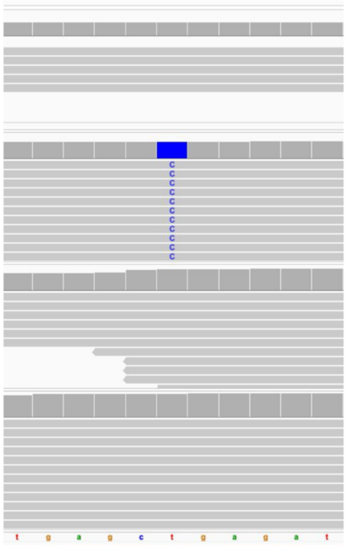

chr20:40497783

(G)

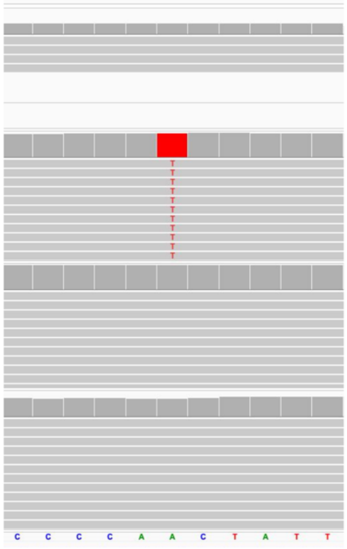

chr20:41962110

(H)

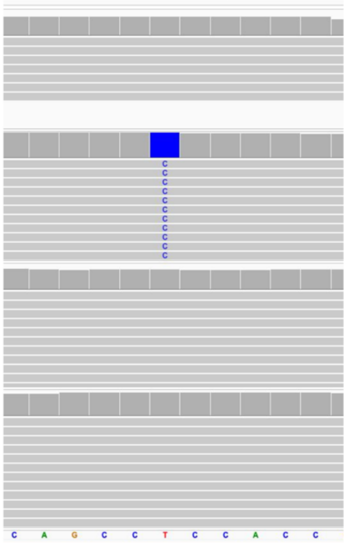

chr20:48686938

(I)

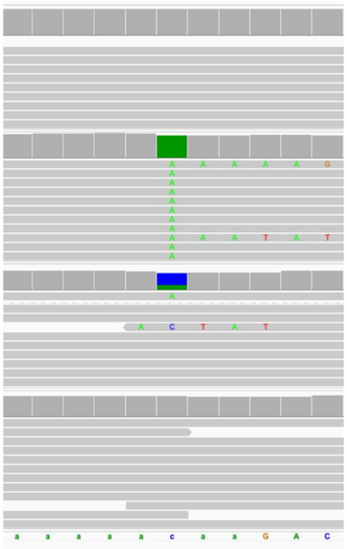

chr20:37952604

(J)

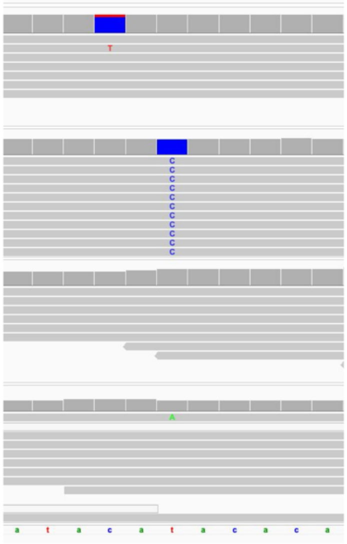

chr20:39012197

(K)

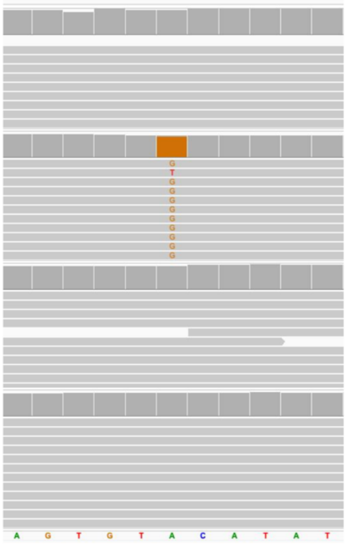

chr20:39146268

(L)

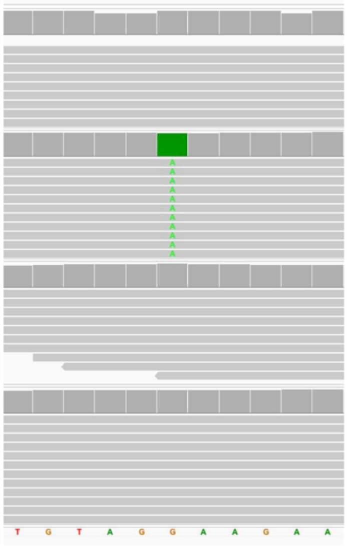

chr20:10710848

(M)

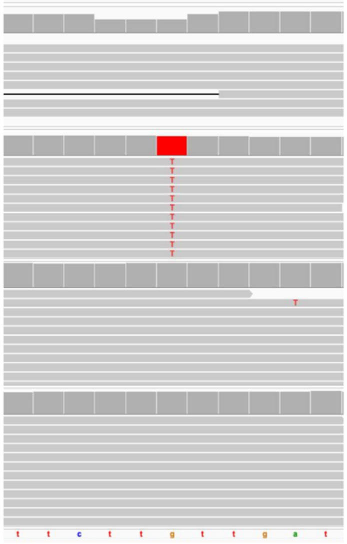

chr20:11364014

(N)

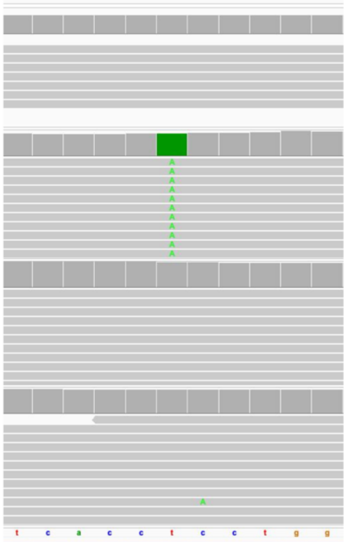

chr20:35400015

(O)

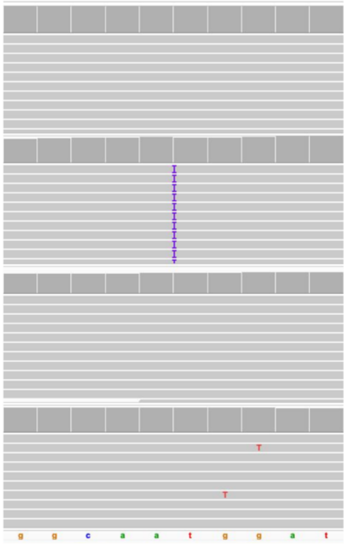

chr20:4962771

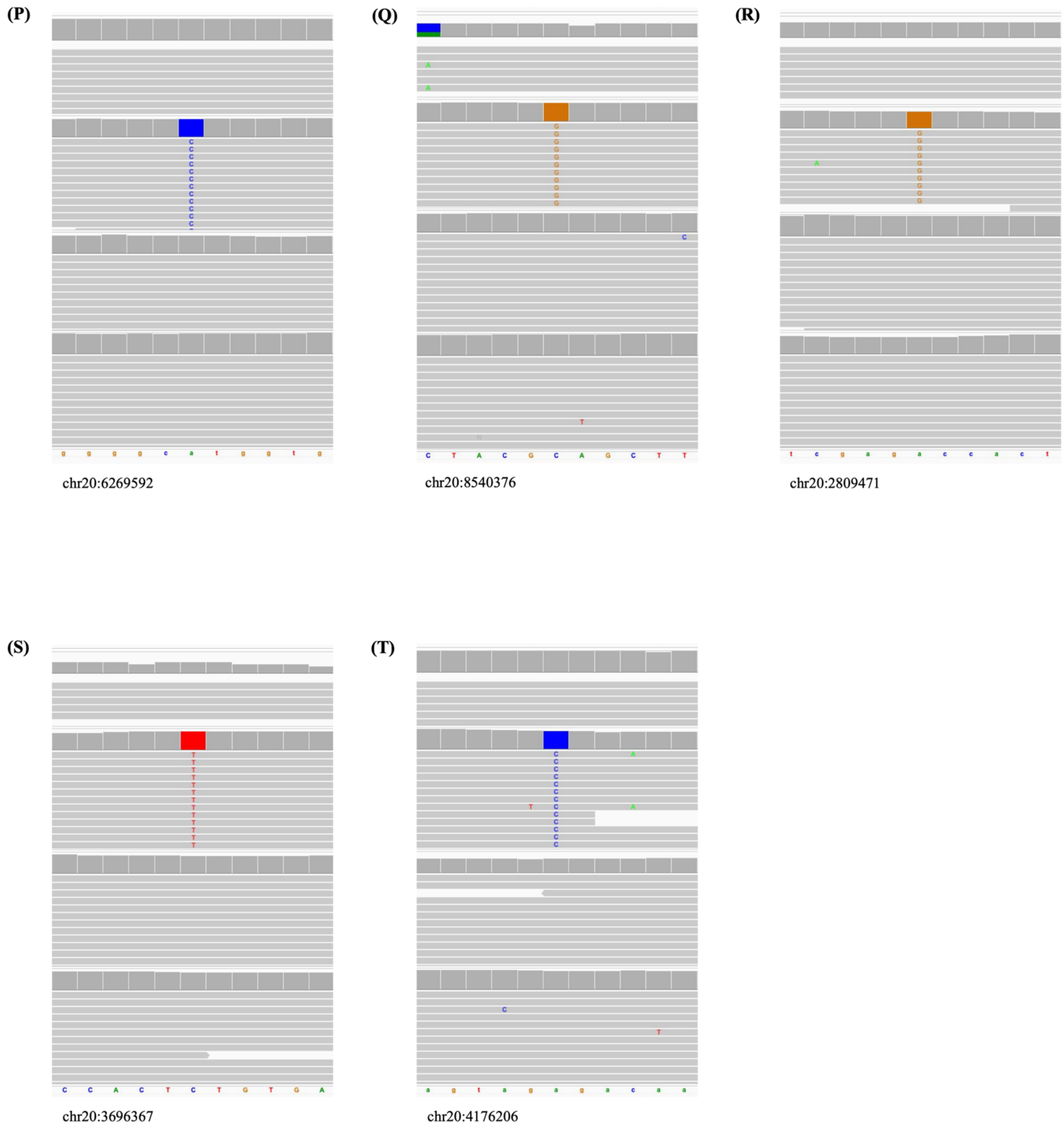

Supplementary Figure 2. Representative Quality score (Q-score) of the ONT long read sequencing run. Sequencing has been performed using flowcell FLO-PRO002 (R9.4.1) on a PromethION P-24 device with SQK-MLK111-96-XL barcoded ligation library preparation kit. Axis X represents sequencing run time span (hours:minutes). Axis Y represents the Q-score of the generated reads, with the solid line indicating the most frequent quality score of reads in the run at the given moment. Blue shaded area indicates the spread of quality scores. Red shaded area represents rejected low Q-scores.

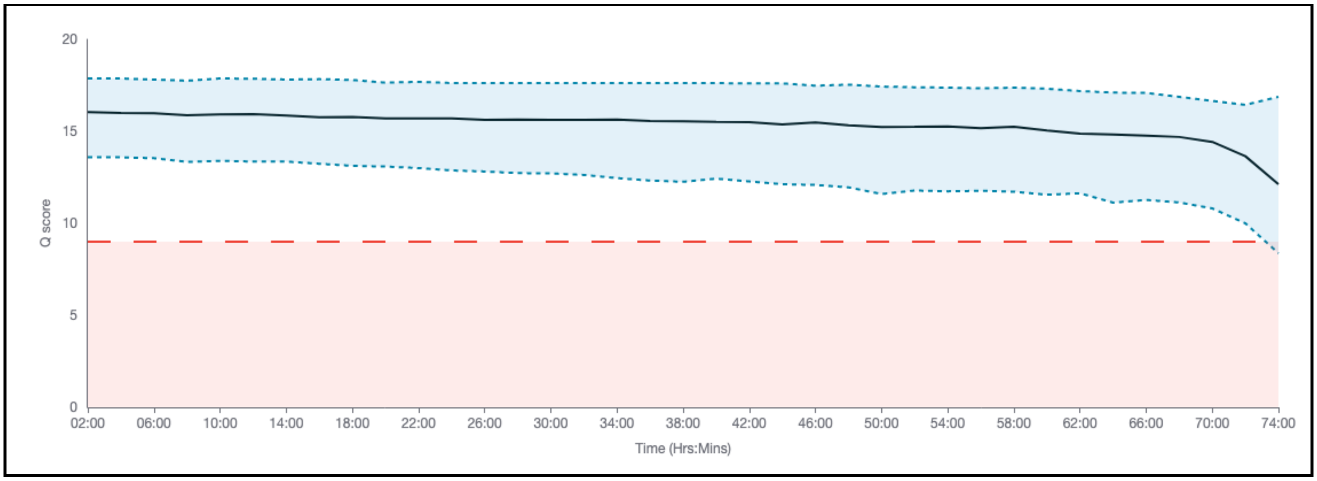

Supplementary Figure 3. STR visualization supporting tool. Heterozygous DMPK expansion in NA05164 sample is demonstrated in long read ONT sequencing utilizing STR visualization tool (https://github.com/epi2me-labs/wf-human-variation) integrated with Variantyx analytical process. This approach serves as a supporting STR evaluation aid utilized in addition to the inspection of the reads' sequences. Axis X represents tandem repeat number, and axis Y represents a number of the reads supporting the corresponding repeat length. Blue shaded area represents repeats in the normal range while red shaded area represents pathogenic repeat expansion range.

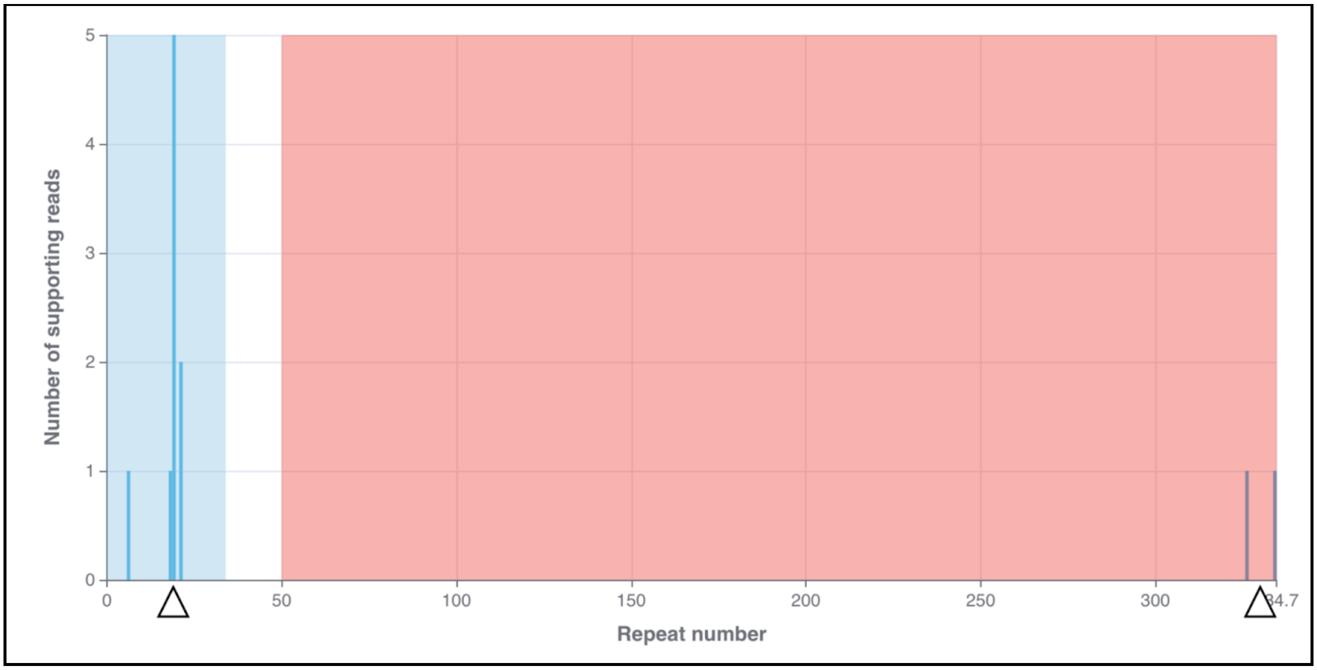

Supplementary Figure 4. Distribution of the average depth of coverage. Depth of coverage of different chromosomes is expressed as a ratio of global depth of genomic coverage. Error bars represent standard deviation (SD). This graph displays autosomes only: sex chromosome coverage is naturally affected by chromosomes copy number and is utilized with appropriate normalization,

while mitochondria coverage is dramatically different from genomic coverage due to high and variable mitochondria abundance, and cannot be accounted for in the depth model.

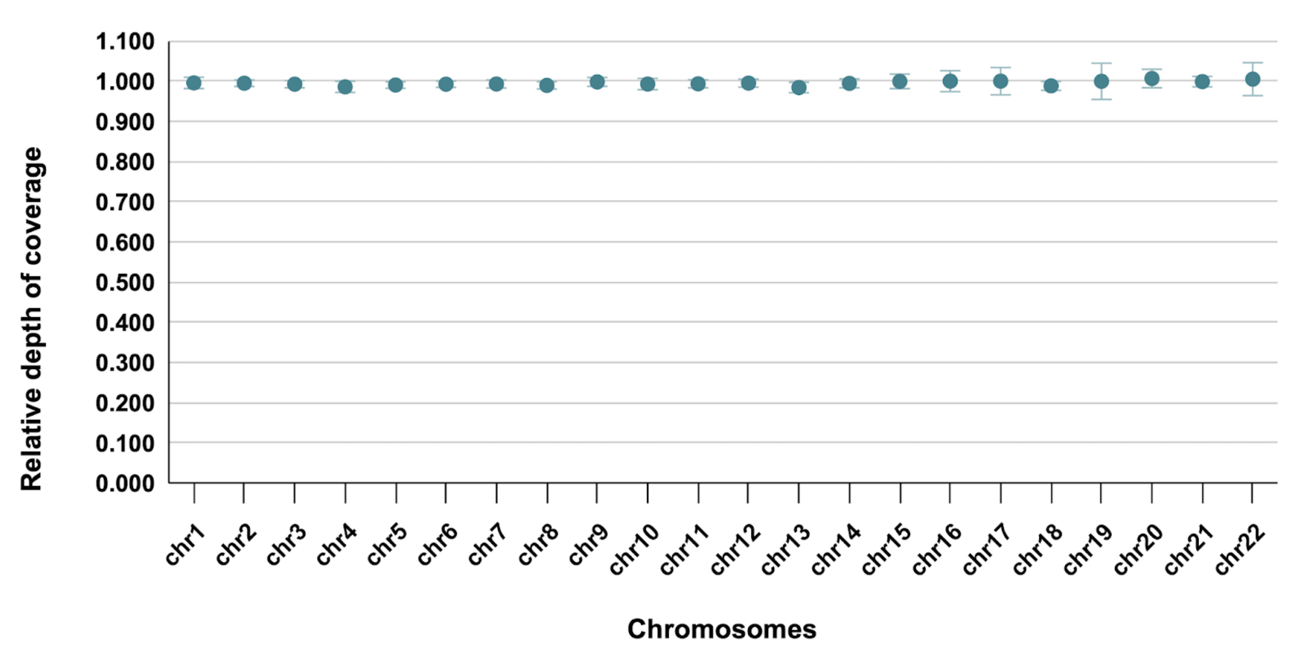

Supplementary Table 1. Samples and variant types

Sample ID*	Purpose
Genome in a Bottle (GIAB) samples: NA12878, NA24385	Analytical validation: Small sequence Changes (SSC) up to 50 bp (Including Mitochondrial Variants) and structural variants (SV)
NA03251, NA01361, NA11906, NA11605, Sample_1, Sample_2, Sample_3, Sample_15	Clinical detection confirmation - Small Sequence Changes (SSC) up to 50 bp (Including Mitochondrial Variants)
Copy Number Variants (CNVs): NA04372, NA05123, NA06047, Sample_4, Sample_5, Sample_6, Sample_7, Sample_8, Sample_9, Sample_10, Sample_11, Sample_13	Clinical detection confirmation - Structural Variants (SVs)/Copy Number Variants (CNVs) over 50 bp

Short Tandem Repeats (STRs): NA03561, NA13515, NA13503, NA13716, NA23709, NA06926, NA06151, NA05164, NA03759, NA23378, NA16213, NA15850, NA06897, NA09237, NA20239, NA07175, Sample_12<br><br>Mobile Element Insertion (MEI): NA11601, NA13805<br><br>Uniparental disomy (UPD): Sample_15	

*Sample IDs starting with NA indicate samples that were purchased via Coriell repository (Coriell Institute, n.d.). IDs Sample_1-15 represent de-identified clinical samples.

Supplementary Table 2. Typical ploidy detected in a 46 XX female sample without aneuploidies (Sample_13).

Chromosome	Predicted Ploidy	Normalized Ploidy	Read Coverage
chr1	2	1.99	11.27
chr2	2	2.01	11.42
chr3	2	2.02	11.45
chr4	2	2.02	11.48
chr5	2	2.01	11.39
chr6	2	2.01	11.41
chr7	2	2.0	11.32
chr8	2	2.01	11.4
chr9	2	2.0	11.41
chr10	2	1.99	11.24
chr11	2	2.0	11.33
chr12	2	2.0	11.32
chr13	2	2.02	11.46
chr14	2	1.99	11.29
chr15	2	2.0	11.32

chr16	2	1.98	11.16
chr17	2	1.95	10.98
chr18	2	2.01	11.43
chr19	2	1.93	10.73
chr20	2	1.99	11.37
chr21	2	1.99	11.39
chr22	2	1.95	11.09
chrX	2	2.01	11.35
chrY	0	0.0	0.03

Supplementary Table 3. Typical ploidy detected in a 46 XY male sample without aneuploidies (Sample_5).

Chromosome	Predicted Ploidy	Normalized Ploidy	Read Coverage
chr1	2	1.99	10.5
chr2	2	2.0	10.61
chr3	2	2.01	10.64
chr4	2	2.01	10.64
chr5	2	2.01	10.65
chr6	2	2.01	10.62
chr7	2	2.01	10.64
chr8	2	2.0	10.59
chr9	2	2.0	10.63
chr10	2	1.99	10.53
chr11	2	2.01	10.6
chr12	2	2.0	10.54
chr13	2	2.01	10.67
chr14	2	2.0	10.58

chr15	2	1.99	10.47
chr16	2	1.99	10.45
chr17	2	1.96	10.26
chr18	2	2.01	10.63
chr19	2	1.95	10.12
chr20	2	1.99	10.56
chr21	2	2.0	10.64
chr22	2	1.95	10.31
chrX	1	1.0	5.3
chrY	1	0.98	5.33

Supplementary Table 4. Representative ONT long-reads variant calling metrics in NA24385 as evaluated against the corresponding GIAB truth set.

Variant Type	Recall	Precision	F1
**SNP**	0.93241	0.96749	0.94963
**Insertions/Deletions <=50bp**	0.44933	0.74429	0.56036
**SV**	0.79167	0.90000	0.84236
**Kit type**	SQK-MLK111-96-XL		
**Flow cell type**	FLO-PRO002		
**Coverage depth**	11.07		

Supplementary Table 5. Standard operation procedure (SOP) for orthogonal variant confirmation with long reads sequencing at Variantyx. Variants previously detected with different technology are verified with ONT long read sequencing. Access to all visualization and analytical tools is governed by the proprietary Variantyx diagnostic console. Analysis is performed following a two-layer approach where variants are analyzed by variant specialists and then reviewed by additional specialists (in case of clinical implementation the final report is signed by the laboratory director).

Variant type	Instructions	Interpretation algorithm		
		**Confirmation criteria**	**Read coverage criteria**	**Reporting and/or Further action**
Small sequence changes (SSC): single nucleotide variants and insertions / deletions/combinations of thereof up to 50bp	Access IGV variant visualization tool with coordinates of the analyzed variant.<br><br>Count the reads supporting the variant and assess the local depth of coverage.	Variant is supported by 2 reads or above	-	Variant is orthogonally confirmed
		Variant is NOT supported by 2 reads or above	Local coverage >= 12x	Variant is NOT confirmed.
		Variant is NOT supported by 2 reads or above	Local coverage < 12x	No conclusion, additional sequencing of the sample should be performed
Copy number variants below 100 kbp	Access IGV variant visualization tool with coordinates of the analyzed variant.<br><br>Count the reads supporting the variant and assess the local depth of coverage.<br><br>Soft-clipped reads that align over both sides of the breakpoint can be included.	Variant is supported by 2 reads or above	-	Variant is orthogonally confirmed
		Variant is NOT supported by 2 reads or above	Local coverage >= 12x	Variant is NOT confirmed.
		Variant is NOT confirmed by 2 reads or above	Local coverage < 12x	No conclusion, additional sequencing of the sample should be performed
Mobile element insertions (MEI)	Access IGV variant visualization tool with coordinates of the analyzed MEI variant. Look for insertions that correspond to the MEI in question. The exact position of the insertion might vary over a region of several nucleotides.	Variant is supported by 2 reads or above	-	Variant is orthogonally confirmed
		Variant is NOT supported by 2 reads or above	Local coverage >= 12x	Variant is NOT confirmed.
		Variant is NOT confirmed by 2 reads or above	Local coverage < 12x	No conclusion, additional sequencing of

	Count the reads supporting the variant and assess the local depth of coverage.<br><br>Get blastn results of the variant and verify the MEI type.			the sample should be performed
Inversions	Access IGV visualization tool with coordinates of the left and right breakpoints of the inversion in question.<br><br>Count the reads supporting the variant and assess local depth of coverage.<br>Include the soft-clipped reads that align over both sides of the breakpoint of the variant. The two parts of the soft clipped reads should be aligned in opposite directions.	Variant is supported by 2 reads or above	-	Variant is orthogonally confirmed
		Variant is NOT supported by 2 reads or above	Local coverage >= 12x	Variant is NOT confirmed.
		Variant is NOT confirmed by 2 reads or above	Local coverage <12x	No conclusion, additional sequencing of the sample should be performed
Copy number variants above 100 kbp	If the exact breakpoint coordinates are available, the same method as described for copy number variants below 100 kbp can be used.<br><br>If the exact coordinates of the breakpoints are not determined, the variant should be examined using SVplot depth visualization tool.<br><br>Deletions should have a drop in coverage from 1 to 0.5 (heterozygous) or to 0 (homozygous). Duplications should have an increase from 1 to 1.5 and triplications should have an increase from 1 to 2.	The expected gain or loss in depth is observed.	Global average coverage over the genome >= 8x	Variant is orthogonally confirmed
		The expected gain or loss in depth is NOT observed.	Global average coverage over the genome >= 8x	Variant is NOT confirmed.
		-	Global average coverage over the genome <8x	No conclusion, additional sequencing of the sample should be performed
Uniparental disomy (UPD)	The variant is analyzed by a cumulative analysis of multiple small sequence	At least 95% of the inspected variants are not	At least 10% of the	The UPD Variant is

	changes (SSC) in the UPD region.<br>The source of UPD (maternal, paternal) should be established prior to the long-reads based verification.<br><br>SSC variants matching the following criteria should be selected for inspection: present as homozygous alt in the parent not represented in the UPD region, while absent in the parent that is a source of UPD. Selection of the variants should be performed based on the short reads sequencing of the parents.<br><br>UPD verification is performed by confirming that those variants are not observed in the proband's long reads sequencing.<br><br>At least 20 SSC, matching those selection criteria and distributed throughout the UPD region, should be inspected. Assess each SSC as indicated for single SSCs	observed in the proband	inspected residues have coverage >= 12x.	orthogonally confirmed
		More than 5% of such inspected variants are observed in the proband on more than 2 reads each.	-	The UPD Variant is NOT confirmed
		-	Less than 10% of the inspected residues have coverage >= 12x.	No conclusion, additional sequencing of the sample should be performed
Aneuploidies	Access aneuploidy tool and review the ploidy and depth of coverage. Ploidy numerical output reflects read coverage distribution over the genome.	Normalized ploidy support the variant	Global average coverage >= 8x	Aneuploidy is orthogonally confirmed
		Normalized ploidy does NOT support the variant	Global average coverage >= 8x	Aneuploidy is NOT performed
		-	Global average coverage < 8x	No conclusion, additional sequencing of the sample should be performed

Mitochondrial variants	Access IGV variant visualization tool with coordinates of the analyzed variant.<br><br>Count the reads supporting the variant and assess the local depth of coverage.	Variant is supported by 10 reads or above	-	Variant is orthogonally confirmed
		Variant is NOT supported by 10 reads or above	Local coverage >=1000x	Variant is NOT confirmed.
		Variant is NOT confirmed by 10 reads or above	Local coverage <1000x	No conclusion, additional sequencing of the sample should be performed
Short Tandem Repeat (STRs)	Access IGV variant visualization tool with coordinate ranges of the analyzed STR.<br><br>Look for insertions or deletions that correspond to the STR variant in question. Get the number of repeats, considering the repeat unit of this STR.<br><br>For repeat expansions that do not have at least two reads encompassing the whole repeat, inspect clipped reads at the STR region and count the observed number of repeats.<br><br>Expanded STRs are subject to somatic variability. Report the observed range.	STR variant range is supported by 2 reads or above	-	STR variant is orthogonally confirmed
		STR variant range is NOT supported by 2 reads or above	Local coverage >=12x	STR variant is NOT confirmed
		STR variant range is NOT supported by 2 reads or above	Local coverage <12x	No conclusion, additional sequencing of the sample should be performed

Supplementary Table 6. Short Tandem Repeats.

STR	Reference repeat count	Coordinates
AFF2 (CGG)	20	chrX:148500631-14850069

AFF3 (CGG)	8	chr2:100104798-100104822
AR (CAG)	23	chrX:67545316-67545385
ATN1 (CAG)	19	chr12:6936716-6936773
ATXN1 (CAG)	30	chr6:16327633-16327723
ATXN10 (ATTCT)	14	chr22:45795354-45795424
ATXN2 (CAG)	23	chr12:111598949-111599018
ATXN3 (CAG)	11	chr14:92071009-92071042
ATXN7 (CAG)	10	chr3:63912684-63912714
ATXN8OS (CTG)	15	chr13:70139383-70139428
BMPR2 (*)	12	chr2:100104798-100104822
C9orf72 (G4C2)	33	chr9:27573528-27573546
CACNA1A (CAG)	13	chr19:13207858-13207897
CBL (*)	11	chr11:119206289-119206322
CNBP (CCTG)	20	chr3:129172576-129172656
CSTB (C4GC4GCG)	3	chr21:43776443-43776479
DIP2B (CGG)	7	chr12:50505001-50505024
DMPK (CTG)	20	chr12:50505001-50505024
FMR1 (CGG)	20	chrX:147912050-147912110
FXN (GAA)	6	chr9:69037286-69037304
HTT (CAG)	19	chr4:3074876-3074933

JPH3 (CTG)	14	chr16:87604287-87604329
NOP56 (GGCCTG)	4	chr20:2652733-2652757
NOTCH2NLC (CGG)	13	chr1:149390803-149390842
PABPN1 (GCN)	10	chr14:23321473-23321502
PHOX2B (GCN)	20	chr4:41745972-41746032
PPP2R2B (CAG)	10	chr5:146878727-146878757
TBP (CAG/CAA*)	37	chr6:170561906-170562017
TCF4 (CTG)	24	chr18:55586155-55586227
